# Using GPS Technology to Understand Spatial and Temporal Activity of Kangaroos in a Peri-Urban Environment

**DOI:** 10.3390/ani8060097

**Published:** 2018-06-17

**Authors:** Timothy Henderson, Karl Vernes, Gerhard Körtner, Rajanathan Rajaratnam

**Affiliations:** 1 Ecosystem Management, University of New England, Armidale NSW 2351, Australia; kvernes@une.edu.au; 2 Zoology, University of New England, Armidale NSW 2351, Australia; gkoertne@une.edu.au; 3 Geography and Planning, University of New England, Armidale NSW 2351, Australia; rrajarat@une.edu.au

**Keywords:** kangaroo, GPS telemetry, movement ecology, activity pattern, peri-urban

## Abstract

**Simple Summary:**

Kangaroo–human conflict is increasing in the peri-urban communities of the New South Wales (NSW) north coast in Australia. A way to assist in managing this conflict is to improve our understanding on the ecology of kangaroos in the peri-urban environment. We utilized modern Global Positioning System (GPS) technology to track adult male kangaroo movements in a peri-urban housing estate at Coffs Harbour, Australia, using both collars and temporary glue-on devices. We also assessed the effectiveness of the glue-on devices, which do not require animal recapture for device retrieval. Kangaroos remained predominately within the residential area and moved over short distances with small movement speeds. Movement activity peaked from 6:00 a.m. to 8:00 a.m., coinciding with daily residential activities such as driving to work, putting children on the school bus, hanging out the washing, and putting bins on the street. In addition, the temporary glue-on devices were effective in gaining information on spatial and temporal activity on a day-by-day basis, despite having short deployment lengths.

**Abstract:**

The increasing kangaroo occurrence in expanding peri-urban areas can be problematic when kangaroos become aggressive towards people and present a collision risk to motor vehicles. An improved understanding on kangaroo spatial and temporal activity patterns in the peri-urban environment is essential to manage kangaroo–human conflict. In this study, we used GPS telemetry to determine activity patterns of male Eastern Grey Kangaroos (*Macropus giganteus*) in a peri-urban community on the north-coast of New South Wales, Australia. Two types of GPS devices were employed; collars and cheaper alternative glue-on units. Kangaroos moved on average 2.39 km a day, with an average movement rate of 1.89 m/min, which was greatest at dawn. The GPS glue-on devices had short deployment lengths of one to 12 days. Despite limitations in attachment time, the glue-on devices were viable in obtaining daily spatial and temporal activity data. Our results aid towards alleviating conflict with kangaroos by providing new insights into kangaroo movements and activity within a peri-urban environment and introduces a potential cheap GPS alternative for obtaining this data relative to more expensive collars.

## 1. Introduction

Understanding the movement patterns of mammals in a peri-urban environment is key to developing appropriate management strategies to mitigate human–animal conflict and facilitate co-existence [1]. Movement patterns of large mammals such as white-tailed deer (*Odocoileus virginianus*) [2,3] and American black bears (*Ursus americanus*) [4], have been studied in North American peri-urban areas. These studies suggest that the urban environment has influenced activity and habitat use of these animals by altering the structure and productivity of resources and introducing movement barriers such as roads and fences [2,3,4].

In Australia, the eastern grey kangaroo (*Macropus giganteus*) is common within the coastal urban communities of the Coffs Harbour Northern Beaches in New South Wales [5]. Peri-urban developments have improved food and water availability, thereby boosting kangaroo numbers [6]. Heritage Park, a residential estate on the Northern Beaches, exemplifies one such community; the estate consists of large, grassy and well-watered residential blocks and supports a density of 1.2 to 1.5 kangaroos per hectare [5]. In this scenario, these densities can be considered high because their numbers substantially influence the frequency of kangaroo-related incidents, including 40 reports of attacks/serious threats by kangaroos in the last 10 years in the Coffs Harbour Northern Beaches region [5,7]. The increase in both human and kangaroo populations in this area necessitates an improved understanding of kangaroo ecology within the peri-urban matrix.

While kangaroo movements have been studied in reserves and farmland [8,9,10], Coulson et al. [11] provided the only published study of kangaroo movements in a peri-urban area. In their study within a golf course and its environs in Anglesea, Australia, tagged eastern grey kangaroos were monitored over six years through radio-tracking, camera trapping and citizen science. This research provided valuable data on long-term population dynamics, seasonal patterns and broad habitat usage by peri-urban kangaroos. However, research on daily kangaroo use of the peri-urban environment remains limited.

Animal movement can be studied through various techniques such as identification tagging, camera trapping, Very High Frequency (VHF) radio-telemetry, and more recently, Global Positioning System (GPS) telemetry [12]. GPS tracking can consistently yield frequent and accurate location estimates, but commercial devices are generally expensive [13]. This price constraint can restrict wildlife research efforts that may be crucial to wildlife management. Allan et al. [14] assessed the use of cheap alternatives to commercial GPS devices, focusing on modified GPS data loggers initially built for recreational purposes. Their study showed the potential of using such devices in understanding the movement ecology of wildlife at a less limiting cost.

In this study, we used two methods of GPS deployment: commercial wildlife GPS collars and cost-friendly off-the-shelf “travel loggers”. We had two main objectives:

(1) To study the spatial and temporal activity of male kangaroos within our peri-urban study site using both types of GPS devices;
(2) To assess the viability of cost-friendly GPS units in providing information on kangaroo movements.

By gaining new insights into kangaroo movements and assessing the effectiveness of cheaper GPS alternatives in obtaining this data, we can aid current and future management of kangaroos in peri-urban environments.

## 2. Materials and Methods

### 2.1. Study Area

**Figure 1:**
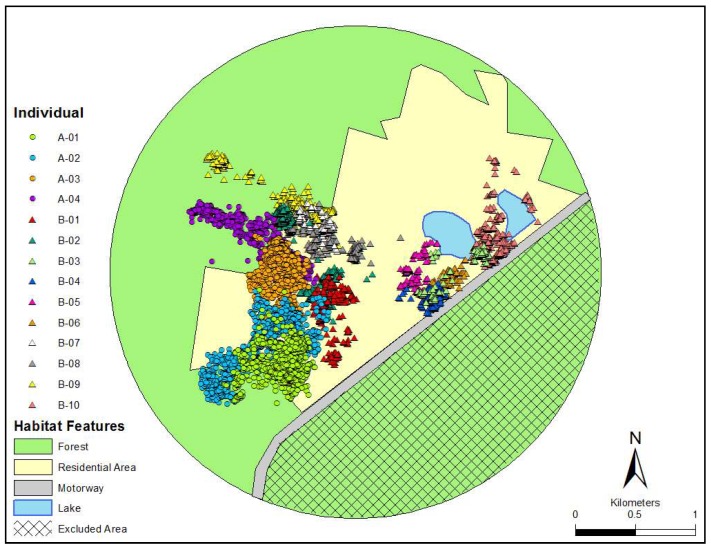
The circle defines the ‘study area’ with location fixes for all individuals, in relation to habitat type (forest and residential area).

Eastern grey kangaroos were studied in the Heritage Park housing estate located on the north-coast of New South Wales, Australia (30°10′42.81″ S, 153°9′6.76″ E). This area currently consists of over 180 properties, which are intermixed with kangaroo habitat, and offer an abundance of grassy spaces, water resources, and shelter. Kangaroos here can be readily observed occupying vacant blocks, as well as in the front and backyards of residential properties. The site is surrounded by a state forest to the north and west, and a motorway to the south-east. As the state forest extended substantially from our study site, we defined a “study area” using a circle large enough to contain all the location fixes for all animals [15], with a 500 m buffer from the outer-most points approximately the diameter of one of the larger kangaroo activity ranges (Figure 1). The motorway served as a boundary for kangaroo movement, therefore the area to the south-east of the motorway (approximately 348 ha) was not included in any analysis. The rest of the site included approximately 415 ha of urban-defined area (excluding 23 ha of lakes) and approximately 485 ha of state forest. Although the study was primarily undertaken during the Australian winter (June to August), food here is not limited due to the availability of well-managed lawns. There was also no distinct evidence of seasonal breeding as pouched young were observed all-year-round.

### 2.2. GPS Telemetry

Two methods of GPS deployment were used. (1) We used four commercial GPS/VHF collars (Sirtrack Wildlife Tracking Solutions, Havelock, New Zealand) weighing approximately 120 g each. GPS collars gathered detailed, long-term data on kangaroo movements, but required the animal to be recaptured for retrieval. (2) We used six cheaper purpose-built ‘glue-on’ drop off GPS/VHF devices on 10 additional kangaroos. These devices were made by combining an off-the-shelf i-gotU GT-120 GPS ‘travel logger’ (Mobile Action Technology, Taipei, Taiwan) weighing approximately 20 g each, with a VHF transmitter (~40 g; Sirtrack). To protect the device, the GPS and VHF components were packaged by heat-shrink to waterproof, with the total weight being approximately 60 g. Fallen devices were retrieved by homing in on the VHF signal once the device was deemed to have dropped off. Although glue-on devices were expected to have shorter deployment times, they were deployed to increase sample size and to assess their effectiveness as a cheaper alternative to expensive collars.

The cost for GPS collars was approximately AUD$2500 per collar. Cost for a glue-on device was approximately AUD$250 ($70 for the i-gotU GT-120, $170 for a VHF transmitter, and $10 for additional items). Both devices were programmed to record a location fix every 15 min. GPS collars were additionally programmed to only record a fix if the horizontal dilution of precision (HDOP) was <3 as a lower HDOP value indicates a more accurate fix [16]. However, i-gotU GPS devices did not have this feature causing some units to record several erroneous fixes. These were identified and deleted using excessive distances and turning angles close to 180 degrees as criteria.

### 2.3. Capture and Handling

We selected large adult male kangaroos because they are of great concern with respect to negative interactions with people. In addition, due to concerns over the welfare of females and their dependent young, female kangaroos were not included in this study. Individual males were selected on a vacant block or on a spacious open property with adequate space for safe capture and recovery. We first obtained verbal permission from property owners to capture and handle individuals within their properties.

Individuals were captured using a Pneudart X-Caliber rifle loaded with a tranquilizing dart containing the sedative drug Zoletil. Dosage varied (200–300 mg) depending on the estimated size of the individual. A licensed shooter operated the rifle, firing from either a vehicle or on foot when appropriate. Due to the tolerance of resident kangaroos towards human presence, individuals were safely darted at a range of 10 to 15 m. Darted individuals were observed from a safe distance until sedated in a lateral recumbent position. Time until sedation varied between individuals, with some taking only a few minutes and others up to 15 min.

**Figure 2:**
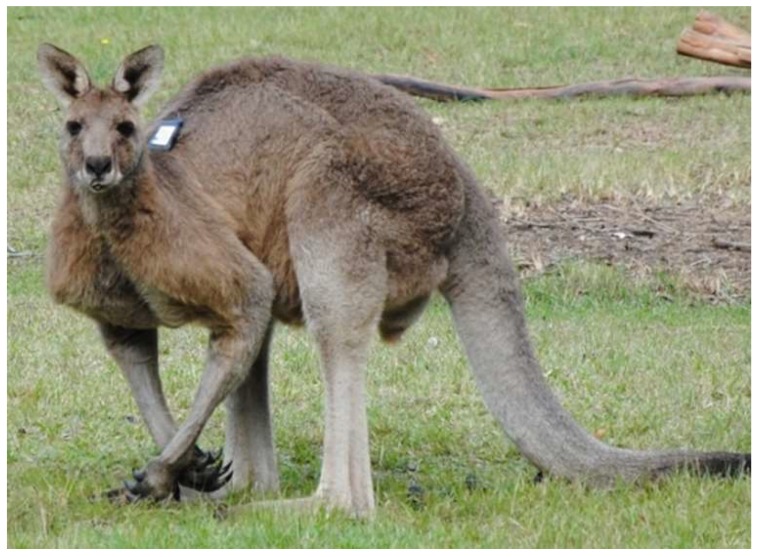
The position of the glue-on i-gotU GPS device on an individual. As seen, positioning the device just below the shoulders removes the stress placed on the adhesive when the kangaroo bends its neck and shoulder.

Individuals fitted with GPS collars required short handling times due to the ease of attachment. Collars were attached with suitable tightness around the neck to reduce movement but not cause stress or irritation. When attaching the glue-on devices, an electric shaver was first used to create a device-sized patch (retaining about 1 cm of underfur) on one side of the individual’s spine between their shoulders to prevent self-removal (Figure 2). Surgical adhesive was applied to the back of the device, and the device was held on the shaved patch for about 30 s until the adhesive set. Total handling time was approximately 5–10 min per individual. After handling, animals were observed from a safe distance until full recovery. Recovery times (from initial sedation to actively walking again) ranged from two to four hours. Due to the large size of the animals, and to reduce additional handling complications, animals were not weighed. This limitation meant we could not use body mass as a variable between individuals. Animal capture and handling was approved by the University of New England Ethics Committee (AEC16-027) and the National Parks and Wildlife Services Scientific License (SL10172).

### 2.4. Data Analysis

To determine habitat preference (forest versus residential area) we used the habitat composition of our defined ‘study area’ (Figure 1) following similar methods used by Körtner et al. [15]. This figure was created in ArcGIS and a 100% Minimum Convex Polygon (MCP) was calculated for each individual using the Animal Movement V2 extension for Arcview 3.2, to provide an extent of the area used [17]. We then used Jacobs’ Index [18] to measure habitat selection using the formula: *D =* (*r* − *p*)/(*r* + *p* − *2rp*), where *r* is the proportion of habitat used and *p* is the proportion of habitat available. This provides a value *D*, which varies from −1 (strong avoidance) to +1 (strong preference), with values close to zero indicating that habitat use is in proportion to availability. Movement data, including incremental MCP, daily distance moved, speed, turning angles as well as the distance of all recorded locations to houses were calculated with programs written using Visual Basic 6 (Microsoft, Redmond, WA, USA). Incremental MCP was used to ascertain the point (in days) at which an individual’s MCP had reached a plateau. Daily distance moved was the sum of all distances between location records over one day. Daily distances were then averaged for each individual and for each GPS type. Speed was calculated in m/min and separated into activity and rest using a “speed threshold” of 5 m/min. Speed threshold was chosen over distance to account for the slight variation in the interval between location fixes. We used the change in angle between consecutive fixes to determine directional behavior of individuals [19]. To describe movement patterns, a circular-angular correlation was used to assess the relationship between distance traveled and turning angle. All statistical analyses were conducted using the software R. Individuals tracked for less than three days were excluded from the ‘daily distance’ averages and statistical analyses.

## 3. Results

We obtained GPS data on 14 male kangaroos. Four GPS collared individuals recorded 63 days of data each with an average of 4239 ± 38 location fixes at a 70 ± 0.6% fix success rate (Table 1). Median HDOP values ranged from 1.8 to 1.9. The deployment of glue-on devices ranged from 1–12 days (average of 4.4 ± 1 day). Fix rate success was >89% but because these devices could not record HDOP, their fix reliability is unknown. The MCP ranging area for collared individuals ranged from 31 to 68 ha, while individuals with glue-on devices ranged from 6 to 63 ha (Table 1). Incremental MCP analysis on the four GPS collared individuals revealed that 42 days were sufficient to ascertain a reliable estimate of an individual’s ranging area because all four had achieved 96% of their final MCP at this mark.

Only six of the 14 kangaroos were recorded in the forested area of the site and their Jacobs’ index suggested avoidance of the habitat type (Figure 2; Table 1). Furthermore, kangaroos had a relatively high occurrence close to houses. Collared individuals and individuals with i-gotU devices recorded on average 42 ± 6% and 60 ± 9.5% of their location fixes within 50 m of a house, respectively.

**Figure 3:**
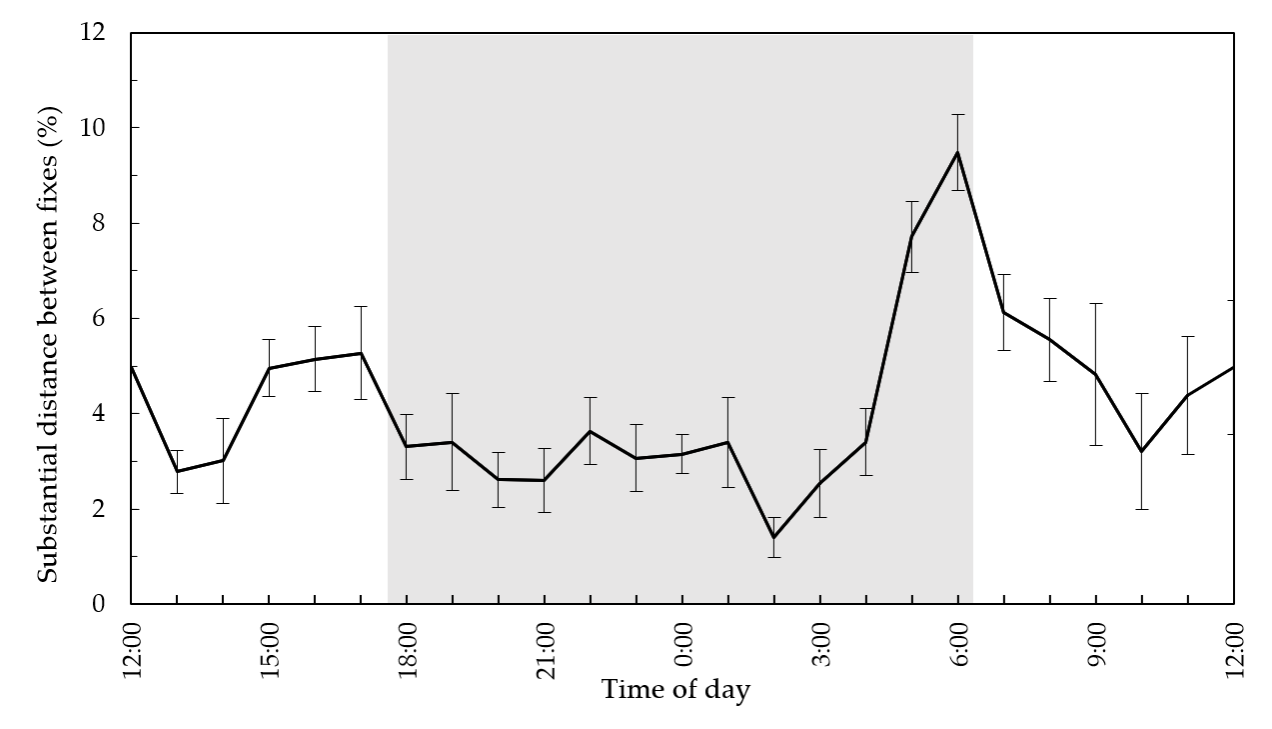
Diel substantial movement activity (with standard error bars) for all studied eastern grey kangaroos at Heritage Park, Coffs Harbor, New South Wales, Australia. Note: Substantial movements refer to a speed threshold that we considered greater than 5 m/min. The shaded area represents the nocturnal period.

Kangaroos moved on average 2.39 ± 0.62 km per day (accumulative distance between location fixes over a day) with the maximum distance moved by an individual on a single day being 4.39 km (Table 2). There was no significant difference in the average daily distance moved between collared and glue-on fitted individuals (f = 1.66, df = 1, *p* = 0.20). The average speed for kangaroos was 1.89 ± 2.31 m/min (Table 2). However, average speed was significantly higher for collared individuals than glue-on fitted individuals (f = 5.15, df = 1, *p* < 0.05). Speed of travel and turning angle were significantly correlated (f = 18.45, df = 1, *p* < 0.001). The maximum of the sin-function at 19.08 degrees indicates that higher speed values represent more directional movement. Kangaroos showed an increase in movement activity around dawn with a peak at 6:00 am where almost 10% of substantial movements (>5 m/min) occurred (Figure 3).

## 4. Discussion

The expansion of peri-urban communities and increasing eastern grey kangaroo populations in Eastern Australia has led to kangaroos often sharing the peri-urban environment with humans, which can lead to kangaroo–human conflict [5]. The first objective of our study was to investigate the spatial and temporal activity of kangaroos in a peri-urban environment to assist with managing kangaroo–human interactions. Kangaroos in our study showed avoidance for the state forest surrounding the study site, remaining predominately among the residential areas. Additionally, a motorway with a wildlife fence runs adjacent to the site and appears to barrier kangaroo movement. The high proportion of fixes close to housing emphasizes the importance of urban green space (i.e., lawns and vacant blocks) to peri-urban kangaroos due to the high availability of resources and open spaces [11].

Kangaroos at our study site were mostly sedentary with a few large-scale movements. Coulson et al. [11] reported similar findings in a peri-urban kangaroo population in Anglesea, Australia. As the duration of our study was short, it is unknown if movements are seasonally influenced. The distance moved by kangaroos in this study were small [8] at approximately two km a day with low movement speeds. These further highlight the high-quality habitat found in the peri-urban matrix, allowing individuals to obtain food, water, shelter, and to undertake other fitness-enhancing activities within a relatively small area. Additionally, their diel movement rates showed a clear peak in longer distance movements around dawn. However, other studies on eastern grey kangaroo activity showed increases in foraging activity at dawn and dusk [20,21,22]. While a peak in dawn movement activity is consistent with these other studies, it is uncertain as to why no peak occurred at dusk. Although our results are not directly comparable due to differences in the methods (e.g., activity type and season), it is possible that a more consistent diel activity is a trait of peri-urban kangaroos which requires further research.

From the perspective of managing human–wildlife conflict, a preponderance by kangaroos to frequently occur close to houses increases the risk of conflict with people. While many residents welcomed the presence of kangaroos on their lawns and in the immediate neighborhood [23], the high population density of kangaroos at the site [5] and their activity patterns shown in this study has undoubtedly contributed to the high number of reported kangaroo attacks on people at Heritage Park [7]. The apparent fidelity of individuals to certain areas is important for managing problematic kangaroos as individuals are likely to occur in a very localized area. Furthermore, a peak in activity around dawn by kangaroos (between 5:00 a.m. and 8:00 a.m.) puts animals (and people) at particular risk in terms of kangaroo–vehicle collision. These times also coincide with a high period of activity by human residents as commuters make their way to work and school, and residents hang out washing or put out bins. Our insights into kangaroo activity patterns should assist in ongoing management of potential kangaroo–human interaction through a relevant awareness campaign at Heritage Park. 

The second objective of our study was to assess the two differing GPS telemetry methods in obtaining similar spatial and temporal information. The GPS collars allowed for long-term data acquisition compared to glue-on devices which provided a cheaper option for short-term data, but across a larger number of replicates. The glue-on devices provide the additional benefit of reducing stress on animals by not requiring a recapture to retrieve devices. The cost of these devices also allows them to be used for a diversity of applications, especially in undergraduate or postgraduate research projects with limited budgets. Our study showed that the glue-on devices had short deployment lengths and were insufficient in describing long-term aspects of kangaroo movements. However, the spatial and temporal information achieved by the glue-on devices, was in most cases, comparable to the collars despite the duration gap. This highlights their potential effectiveness as an alternative to commercial collars, depending on the type of data required. 

Our study provided a new insight into kangaroo movement patterns in a peri-urban area, which can be incorporated into localized management decision-making to minimize potential negative interactions with residents. Our information can also be used as an initial benchmark on how kangaroos utilize the peri-urban landscape on a daily basis.

## 5. Conclusions

Increasing sympatric human and kangaroo populations within the human–wildlife dimension of the Australian peri-urban space will pose a management issue well into the 21st century. While our study has provided much needed baseline information on kangaroo spatial and temporal activity in a single peri-urban environment, further research is needed in similar environments elsewhere in Australia. The use of inexpensive glue-on GPS devices can be a viable alternative to long-term GPS collars for obtaining rapid short-term data on kangaroos to guide kangaroo management issues requiring immediate attention.

## Figures and Tables

**Table 1:** Technical details, area of activity, forest usage and selectivity (Jacobs’ index) of individual eastern grey kangaroos at Heritage Park, Coffs Harbor, New South Wales, Australia. Animals beginning with ‘A’ carried GPS collars and those beginning with ‘B’ carried i-gotU GPS devices. Jacobs’ index values provide an indication of selectivity from −1 (strong avoidance) to +1 (strong preference).

Animal	Date Deployed	Duration (days)	Fixes Acquired	Acquisition Rate (%)	MCP (ha)	Fixes in Forest (%)	Jacobs‘ Index
A-01	20 July	63	4256	70	50	4	−0.93
A-02	20 July	63	4133	68	68	17	−0.70
A-03	20 July	63	4312	71	31	<1	−0.98
A-04	20 July	63	4254	70	46	18	−0.68
B-09	28 July	11.5	984	89	63	13	−0.77
B-02	8 June	11.1	1019	95	41	<1	−0.98
B-10	28 July	6.3	586	98	47	0	-
B-08	28 July	6.2	564	95	31	0	-
B-01	8 June	2.8	261	99	20	0	-
B-04	9 June	2.5	236	97	6	0	-
B-03	8 June	2.1	195	97	16	0	-
B-06	9 June	1.5	134	97	7	0	-
B-05	9 June	1.3	121	96	11	0	-
B-07	28 July	1.0	89	94	8	0	-

**Table 2:** The distance moved per day (average and range) and the average speed (including standard deviation) between location fixes for eastern grey kangaroos at Heritage Park, Coffs Harbor, New South Wales, Australia. Animals designated as ‘A’ were fitted with GPS collars while animals designated as ‘B’ were fitted with ‘i-gotU’ glue-on devices.

Animal	Daily Distance (km)		Average Speed (m/min)
	Average	Range	
A-01	2675 ± 569	1480–4245	2.10 ± 2.58
A-02	2549 ± 715	1419–4393	2.09 ± 2.43
A-03	2084 ± 418	1400–3301	1.65 ± 1.72
A-04	2194 ± 421	1403–3520	1.76 ± 2.06
Collar total	2376 ± 594	-	1.90 ± 2.23
B-01	-	2638–3469	2.15 ± 2.27
B-02	2039 ± 551	1088–2706	1.38 ± 2.36
B-03	-	1464–2221	1.65 ± 2.85
B-04	-	1955–2079	1.47 ± 1.76
B-05	-	3108	2.16 ± 2.66
B-06	-	3463	2.45 ± 2.26
B-07	-	2659	1.89 ± 2.08
B-08	2739 ± 454	2182–3310	2.01 ± 3.14
B-09	3106 ± 824	1996–4141	2.19 ± 2.94
B-10	2090 ± 425	1766–2925	1.51 ± 2.29
‘i-gotU’ total	2525 ± 776	-	1.81 ± 2.63
Grand total	2393 ± 618	-	1.89 ± 2.31
